# Comparing Gene Expression Profiles Between Bt and non-Bt Rice in Response to Brown Planthopper Infestation

**DOI:** 10.3389/fpls.2015.01181

**Published:** 2015-12-24

**Authors:** Fang Wang, Duo Ning, Yang Chen, Cong Dang, Nai-Shun Han, Yu'e Liu, Gong-Yin Ye

**Affiliations:** ^1^State Key Laboratory of Rice Biology, Ministry of Agriculture Key Laboratory of Agricultural Entomology, Insect Physiology and Biochemistry, Institute of Insect Sciences, Zhejiang UniversityHangzhou, China; ^2^State Key Laboratory of Rice Biology, China National Rice Research InstituteHangzhou, China

**Keywords:** Bt, brown planthopper, hormone, transcription factors, lipid transfer protein, early nodulin

## Abstract

Bt proteins are the most widely used insecticidal proteins in transgenic crops for improving insect resistance. We previously observed longer nymphal developmental duration and lower fecundity in brown planthopper (BPH) fed on Bt rice line KMD2, although Bt insecticidal protein Cry1Ab could rarely concentrate in this non-target rice pest. In the present study, we performed microarray analysis in an effort to detect Bt-independent variation, which might render Bt rice more defensive and/or less nutritious to BPH. We detected 3834 and 3273 differentially expressed probe-sets in response to BPH infestation in non-Bt parent Xiushui 11 and Bt rice KMD2, respectively, only 439 of which showed significant differences in expression between rice lines. Our analysis revealed a shift from growth to defense responses in response to BPH infestation, which was also detected in many other studies of plants suffering biotic and abiotic stresses. Chlorophyll biosynthesis and basic metabolism pathways were inhibited in response to infestation. IAA and GA levels decreased as a result of the repression of biosynthesis-related genes or the induction of inactivation-related genes. In accordance with these observations, a number of IAA-, GA-, BR-signaling genes were downregulated in response to BPH. Thus, the growth of rice plants under BPH attack was reduced and defense related hormone signaling like JA, SA and ET were activated. In addition, growth-related hormone signaling pathways, such as GA, BR, and auxin signaling pathways, as well as ABA, were also found to be involved in BPH-induced defense. On the other side, 51 probe-sets (represented 50 genes) that most likely contribute to the impact of Bt rice on BPH were identified, including three early nodulin genes, four lipid metabolic genes, 14 stress response genes, three TF genes and genes with other functions. Two transcription factor genes, *bHLH* and *MYB*, together with lipid transfer protein genes *LTPL65* and early nodulin gene *ENOD93*, are the most likely candidates for improving herbivore resistance in plants.

## Introduction

Cry proteins isolated from *Bacillus thuringiensis* (Bt) are the most widely used insecticidal proteins worldwide. *Cry* genes have been transferred to many crops to improve their insect resistance, such as cotton, maize, potato, tobacco, rice, soybean, tomato, and eggplant, although some of these crops have not yet been commercialized (Romeis et al., 2006; Saker et al., 2011; James, 2014). The first transgenic rice line harboring a Bt delta-endotoxin gene (under control of the CaMV 35S promoter) was generated in 1989 (Yang et al., 1989). Since then, Bt rice lines expressing *cry* genes, including *cry1Aa, cry1Ab, cry1Ac, cry1Ab/Ac, cry1C*, and *cry2A*, have been developed and have undergone various stages of testing (Chen et al., 2011). Bt rice lines, such as KMD, T1c-9, T2A-1, were reported to effectively control target Lepidoptera insects such as stem borer and leaf folder (Ye et al., 2001, 2003; Chen et al., 2005; Zheng et al., 2011; Wang et al., 2015). As Bt protein is toxic to target pests, its potential effects on the environment have attracted widespread attention, especially its influence on the food safety and ecological security of non-target organisms (O'Callaghan et al., 2005; Chen et al., 2006; Wang et al., 2006; Yu et al., 2011). The potential risks of Bt rice to arthropod communities, non-target herbivores, predators and parasitoids have been widely assessed. No detrimental effects of Bt rice have been found on most of the assessed arthropods, such as predator spiders *Pardosa pseudoannulata, Ummeliata insecticeps* and *Pirata subpiraticus*, green lacewing *Chrysoperla sinica*, mirid bug *Cyrtorhinus lividipennis* and parasitoid of brown planthopper (BPH) *Anagrus nilaparvatae* (Chen et al., 2009; Gao et al., 2010; Tian et al., 2010, 2012; Han et al., 2014; Li et al., 2014, 2015a). However, significantly longer nymph duration and lower fecundity was found in the non-target herbivores BPH *Nilaparvata lugens* Stål, thrip *Stenchaetothrip biformis* (Bagnall), leafhopper *Nephotettix cincticeps* and ladybird beetle *Propylea japonica* (Thunberg) feeding on Bt rice in laboratory experiments (Akhtar et al., 2010; Chen et al., 2012; Lu et al., 2014; Li et al., 2015a), although no significant reduction in population density was found under field conditions. Li et al. (2015a) attributed the effect of Bt rice on *P. japonica* to unknown differences in the nutritional composition of Bt rice pollen, as it was confirmed that these insects are not sensitive to pure Cry protein. Therefore, Bt-independent variation is thought to exist, which might render rice plants more defensive and/or less nutritious to these insects.

BPH has become the most destructive insect pests of rice in the main Asia-Pacific rice-producing region since the 1970s. To date, 28 BPH resistance loci, including 20 dominant and 8 recessive genes, have been identified from cultivated or wild species of rice; 23 of these genes were mapped to rice chromosome 2, 3, 4, 6, 7, and 12, and only three have been cloned (Du et al., 2009; Cheng et al., 2013; Fujita et al., 2013; He et al., 2013; Tamura et al., 2014; Wu et al., 2014; Liu et al., 2015). However, little is known about the molecular interactions between plants and sucking pests due to the sophisticated behavior of these insects. The response of plants to piercing-sucking pests such as whitefly, aphid and BPH is thought to be similar to the pathogen defense response (Zhang et al., 2004; Yuan et al., 2005; Li et al., 2006; Zarate et al., 2007). Once the pathogen invades the plant, Ca^2+^ influx triggers reactive oxygen species (ROS) production *in situ*, which in turn activates the hypersensitive response in infected cells (Tenhaken et al., 1995). The molecular mechanism of the plant immune response to BPH is not quite clear but is thought to be somewhat similar to the pathogen defense response. Pattern recognition receptors (PRRs) on the cell membrane recognize herbivore- and damage-associated molecular patterns (HAMPs and DAMPs) and thus induce PRR-triggered immunity (PTI, Boller and Felix, 2009; Schulze-Lefert and Panstruga, 2011). PTI, together with the effectors secreted in watery saliva, promotes the basal resistance response, including the activation of salicylic acid (SA), ethylene (ET) and MAPK cascade signaling pathways (Du et al., 2009; Hu et al., 2011; Lu et al., 2011; Cheng et al., 2013). It was hypothesized that jasmonic acid (JA) negatively regulates resistance to the phloem-feeding insect BPH in rice, while the SA and ET pathways positively affect plant resistance to sucking pests (Li et al., 2006; Hao et al., 2008; Zhou et al., 2009; Lu et al., 2011; Tong et al., 2012). Secondary metabolites that deter feeding and inhibit digestion, and plant volatiles that repel herbivores or attract natural enemies, are important components in the interaction between plants and insects. In response to BPH infestation, Ca^2+^ influx can also lead to protein plugging and callose deposition on the sieve (Hao et al., 2008; Hogenhout and Bos, 2011; Bonaventure, 2012), especially in rice carrying *Bph* resistance genes. Volatile organic compounds (VOCs) are useful signals in hosts searching for herbivore insects (Halitschke et al., 2008; Cheng et al., 2013). Meanwhile, the herbivore-induced VOCs also serve as indirect defense signals (Beale et al., 2006).

We previously showed that the Bt insecticidal protein Cry1Ab could be concentrated in *S. bioformis* adults but not in BPH. Although the concentration of Bt insecticidal protein was quite low in BPH, the developmental duration of BPH feeding on Bt rice line Kemingdao 2 (KMD2) was significantly delayed for the first and second generation. Moreover, the fecundity of BPH was significantly lower when fed on Bt rice than on the non-Bt parental plants (Chen et al., 2012). The exact cause of the delayed development and reduced fecundity of non-target herbivores fed on Bt rice remains unknown. In the current study, to investigate the variation in Bt rice that causes changes in BPH performance, we performed microarray (GeneChip) analysis to compare the gene expression profiles between Bt rice and non-transgenic parental plants in response to BPH infestation. The goal of microarray analysis was to detect unintended changes that may have occurred during transformation or tissue culture that have made Bt rice less suitable for feeding and oviposition of the non-target insect pest BPH.

## Materials and methods

### Plant materials

Bt rice line KMD2, which is highly resistant to stem borer and was developed using *Agrobacterium*-mediated methods, was used in this experiment, along with its untransformed parental *japonica* cultivar Xiushui 11. The Bt rice line expresses the insecticidal protein gene *Cry1Ab* under the control of the maize *ubiquitin* promoter, which is linked in tandem with *gus* (encoding β-glucuronidase), *hpt* (encoding hygromycin phosphotransferase) and *npt* (encoding neomycin phosphotransferase) (Ye et al., 2001). A total of 200 uniform seeds per line were soaked in deionized water at 25°C for 2 days, germinated on a plastic board covered with plastic film at 35°C for 1 day and grown in a controlled chamber at 30°C in the light and 25°C in the dark under a 16:8 h light: dark regime. The relative humidity was maintained at 85%. Three weeks later, rice seedlings of similar sizes were transplanted into glass tubes (38 × 250 mm) covered with nylon mesh, with one tube per seedling. The glass tube was filled with 5 ml nutrient solution, which was renewed every 3 days (Akhtar et al., 2010).

For BPH treatment, 10 s-instar nymphs were infested onto each 30-day-old seedling. More than 30 replicates were prepared for each treatment. After 72 h, the BPH nymphs were carefully removed and rice shoots of both BPH-infested and non-infested plants were sampled for analysis. The BPH colony was originally collected from paddy fields at the Zhejiang University farm in 2008 in Hangzhou, China and was reared on “Taichung Native 1” (TN1) rice (*Oryza sativa* L.) seedlings at 28°C under a photoperiod of 14:10 h (light: dark), as described in Chen et al. (2012).

### RNA extraction and microarray analysis

Frozen rice shoots were homogenized in liquid nitrogen using a mortar and pestle. Three biological replicates were collected for each treatment. Total RNA was extracted using Trizol regent according to the supplier's recommendation (Invitrogen, Karlsruhe, Germany). Residual DNA was removed using an RNeasy MinElute Cleanup Kit (Qiagen). After mixing with poly-A RNA controls, the total RNA was first reverse transcribed using T7-Oligo^(dT)^ Promoter Primer for the first-strand cDNA synthesis reaction. Following RNase H-mediated second-strand cDNA synthesis, the double-stranded cDNA was purified and served as a template in the subsequent *in vitro* transcription reaction, which was carried out in the presence of T7 RNA polymerase and a biotinylated nucleotide analog/ribonucleotide mix for complementary RNA (cRNA) amplification and biotin labeling. The biotin-labeled cRNA targets were then cleaned up, fragmented and hybridized to the Affymetrix GeneChip 57 K Rice Genome Array according to the manufacturer's protocol. This expression array contains probe-sets to query 51,279 transcripts representing two rice cultivars, with approximately 48,564 *japonica* transcripts and 1260 transcripts representing the *indica* cultivar (Sharma et al., 2012). Expression profiling analysis was carried out in three replications by CapitalBio Corp. (Beijing, China).

### qRT-PCR analysis

An aliquot of purified RNA was reverse transcribed using a first-strand cDNA synthesis kit (Toyobo, Japan), and quantitative real-time PCR was performed using the ABI7500 Real-time PCR Detection System (ABI, Hercules, CA, USA). PCR was performed using SYBR® premix Ex Taq™ with ROX reference dye (Takara, Dalian, China). The PCR conditions consisted of denaturation at 95°C for 30 s, followed by 40 cycles of denaturation at 95°C for 3 s, annealing at 60°C for 34 s. A dissociation curve was generated at the end of each PCR cycle to verify that a single product was amplified. Expression of the target gene was normalized relative to the expression of the housekeeping gene *actin*. The quantification of mRNA levels was based on the method of Livak and Schmittgen (2001). Primers used for qRT-PCR are listed in Supplementary Table S1.

### Quantification of plant hormones by LC-ESI-MS/MS

Samples were prepared according to Pan et al. (2008) and Liu et al. (2012) with minor modifications. Approximately 0.5 g fresh rice shoots for each replicate was ground to a powder in liquid nitrogen, and 4 ml 80% methanol (methanol: water, 80:20, v/v) was added as extraction buffer. Three biological replications were prepared for each treatment. The homogenate was transferred to a 10-ml tube and incubated in a shaker at 100 rpm for 16 h at 4°C. After centrifugation at 10,000 g for 10 min at 4°C, the supernatant was transferred to a fresh tube and concentrated using a nitrogen evaporator with nitrogen flow. Samples were redissolved in 200 μl methanol. Then, 20 μl of sample was injected and analyzed on an Agilent 6460 triple quadrupole LC/MS system (Agilent Technologies, Heilbronn, Germany) outfitted with an electrospray (ESI) source. The hormones were separated by reversed-phase HPLC on a Zorbax XDB C18 column (2.1 × 150 mm, 3.5 μm, Agilent). Separations were performed using a binary solvent system composed of MeOH (solvent A) and 0.1% formic acid in water (solvent B) as a mobile phase at a flow rate of 0.3 ml min^−1^. The elution gradient profile was set as follows: 0–1 min, 40% A + 60% B; 1–6.5 min, 100% A+ 0% B; 6.5–10 min, 100% A+ 0% B. Tandem mass spectrometric analysis was performed in multiple reaction monitoring (MRM) mode. The MRM parameters of each compound are listed in Table 1. Standard chemical regents including IAA (indole-3-acetic acid), GA_1_ (gibberellin A_1_), JA, SA, and ABA (abscisic acid) were purchased from Sigma-Aldrich Chemical Co. (Shanghai, China). The concentration of each plant hormone was calculated using the following formula:

$$\begin{array}{l} \text{Plant hormone}(\text{ng/gFW})={\text{C}}_{\text{standard}}\times {\text{A}}_{\text{sample}}\times {\text{V}}_{\text{sample}}/\\ \;({\text{A}}_{\text{standard}}\times {\text{FW}}_{\text{sample}})\\ (\text{C},\text{ concentration }(\text{ng/ml});\text{ A},\text{ peak area};\text{ V},\text{ volume }(\text{ml});\\ \text{FW},\text{ fresh weight }(\text{g})) \end{array}$$

**Table 1:** **Optimized MRM parameters for the quantification of phytohormones**.

**Analytes**	**Scan mode**	**Transition (m/z)**	**Cone voltage (V)**	**Collision energy (V)**
IAA	+	176.1 → 130.1	75	10
JA	−	209.1 → 59.1	70	2
SA	−	137 → 93	75	10
GA_1_	−	345.2 → 143.1	220	15
ABA	−	263.1 → 153	75	0

Scan mode: + positive, − negative.

### Statistical analysis

The relative expression levels of genes and concentrations of phytohormones were analyzed using Two-way analysis of variance (ANOVA), followed by a Duncan' multiple range significant test. All statistical analysis was performed by the Data Processing System (DPS) package (Version 9.5).

## Results

### Overview of gene expression profiles in response to BPH infestation

**Figure 1:**
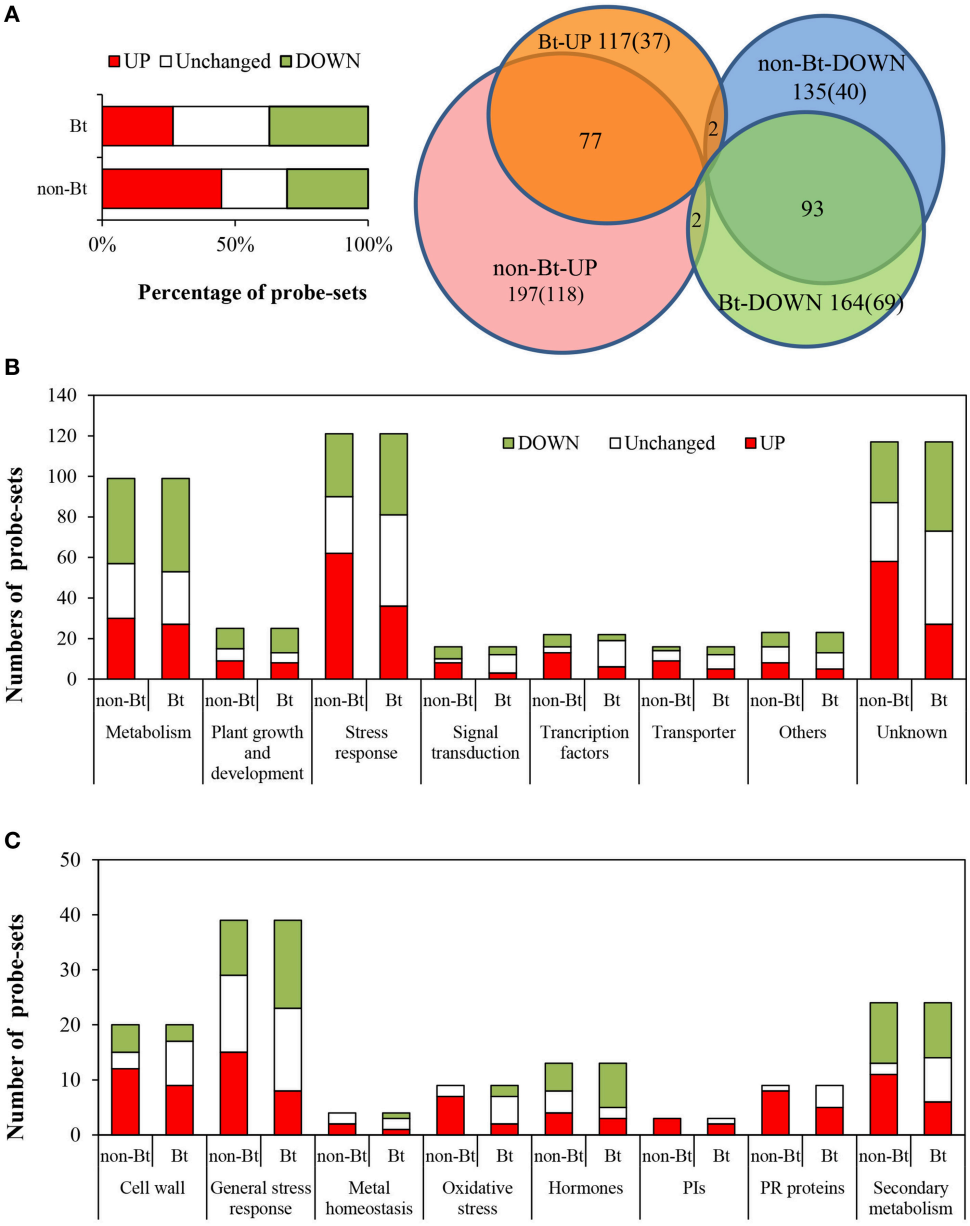
**Analysis of genes with differential responses to BPH between Bt rice and non-Bt rice**. The 439 probe-sets showing differential responses to BPH feeding between Bt rice line and the non-Bt parent were classified into eight categories. **(A)** Left, percentage of upregulated, unchanged, and downregulated genes differing in the response to BPH between Bt rice line and the non-Bt parent; right, Venn diagram. **(B)** Number of up/downregulated probe-sets in each category. **(C)** Number of up/downregulated probe-sets related to stress responses.

Using Affymetrix GeneChip analysis, we found that 21,917 and 21,782 probe-sets were expressed (*P* < 0.05) in non-Bt parental and Bt rice, respectively. The numbers of probe-sets responsive to BPH infestation with different fold-change thresholds, identified using significance analysis of microarray (SAM) methodology, are listed in Table 2. More genes were affected by BPH infestation in non-Bt than in Bt rice. Genes were considered to be differentially expressed at a threshold of 2.0 fold change (FC) up or down (FC ≥ 2.0, upregulated; or FC ≤ 0.5, downregulated; *q*-value < 0.05). When we compared rice shoots infested by BPH for 72 h with non-infested shoots, 3834 and 3273 differentially expressed probe-sets were identified in non-Bt parent and Bt rice line, respectively. Of these, 2589 probe-sets representing 2371 different expressed genes (DEGs) showed similar responses to BPH infestation in the two rice lines, as more than one probe-set corresponds to one gene in some instances (Deveshwar et al., 2011; Supplementary Table S2); the number of downregulated probe-sets was nearly two-fold that of upregulated (907 up/1682 down). There were 1490 probe-sets that only exhibited significant changes in expression in one rice line, but no significant difference were found between two lines as the ratio of FC_Bt rice_ to FC_non−Bt rice_ (data not shown). The impact of BPH attack on the expression of 439 probe-sets (representing 400 DEGs) differed markedly between rice lines (Figure 1A; Supplementary Table S3). Of these, 117 (26.6%) and 164 (37.4%) probe-sets were up- and down-regulated in Bt rice, while there were 197 (44.9%) and 135 (30.8%) probe-sets upregulated and downregulated in non-Bt parent plants, respectively. In addition, there were 107 showed no change in non-Bt parent plants and were up-/down-regulated in Bt rice (Figure 1A).

**Table 2:** **Number of probe-sets responsive to BPH infestation with different fold change thresholds (***q***-value < 0.05) identified using significance analysis of microarray (SAM) methodology**.

**Fold change**	**Up-regulated**		**Down-regulated**		**Total**	
	**non-Bt**	**Bt**	**non-Bt**	**Bt**	**non-Bt**	**Bt**
≥2.0	1534	1269	2300	2004	3834	3273
≥3.0	578	429	1137	967	1715	1396
≥4.0	318	205	744	633	1062	838
≥5.0	200	120	533	452	733	572
≥6.0	131	96	429	350	560	446
≥7.0	102	64	347	290	449	354
≥8.0	84	41	286	241	370	282

### Pathway analysis of genes showing the same response to BPH infestation in Bt and non-Bt rice

Pathway analysis of the 2371 DEGs revealed similar responses to BPH infestation in both rice lines, which was carried out with the Plant MetGenMAP system using the FDR correction method at a threshold of 0.05 (Joung et al., 2009). Altogether, 154 significantly altered pathways were detected, including 43 that were upregulated and 52 that were downregulated. There were also 59 pathways that were either upregulated or downregulated by BPH infestation in both rice lines (Table 3). Plant nitrogen assimilation, chlorophyll biosynthesis, fatty acid biosynthesis and elongation pathways were significantly downregulated in response to BPH feeding. Superoxide radical removal and cell wall modification-related pathways (such as epicuticular wax biosynthesis) were also suppressed. On the other hand, biosynthesis of plant hormones such as IAA and JA was activated by BPH attacking, while salicylate biosynthesis was suppressed. The GA inactivation pathway was upregulated, while ET and brassinosteroid (BRs) biosynthesis pathways were either up- or downregulated. Biosynthesis of amino acids such as asparagine, glutamine and lysine was significantly downregulated, while degradation of arginine, leucine and lysine was upregulated.

**Table 3:** **Pathway analysis of genes showing similar responses to BPH infestation in Bt and non-Bt rice**.

**Categories**	**Pathway name**	***p*****-value**	
		**UP**	**DOWN**
Amino acid and derivative metabolism	Arginine degradation II	0.02291	
	Arginine degradation X (arginine monooxygenase pathway)	0.02291	
	β-alanine biosynthesis II	1.35E-06	
	Citrulline biosynthesis	0.02291	
	Homocysteine and cysteine interconversion	0.02291	
	Isoleucine degradation I	0.02291	
	Leucine biosynthesis	0.02291	
	Leucine degradation I	0.02291	
	Lysine degradation II	0.00105	
	Methionine biosynthesis I	0.02291	
	Methionine degradation III	3.33E-05	
	Phenylalanine biosynthesis I	0.02291	
	Proline biosynthesis I	0.02291	
	Proline biosynthesis II	0.02291	
	Superpathway of citrulline metabolism	0.02291	
	Superpathway of sulfur amino acid biosynthesis (*Saccharomyces cerevisiae*)	0.02291	
	Tryptophan biosynthesis	0.00105	
Carbohydrates metabolism	Acrylonitrile degradation	0.02291	
	Acetyl CoA fermentation to butyrate	0.02291	
	Aldoxime degradation	0.02291	
	D-lactate fermentation to propionate and acetate	1.35E-06	
	Ethanol fermentation to acetate	1.35E-06	
	Ethylene glycol degradation	0.02291	
	Glutamate degradation VII (to butyrate)	0.00105	
	Glutaryl-CoA degradation	0.02291	
	Glycolipid biosynthesis	0.02291	
	Xylulose-monophosphate cycle	3.33E-05	
	Pentose phosphate pathway (oxidative branch)	0.02291	
	GDP-D-rhamnose biosynthesis	0.02291	
	GDP-L-fucose biosynthesis I (from GDP-D-mannose)	0.02291	
	Oxidative ethanol degradation I	3.30E-05	
Plant hormone and secondary metabolites	13-LOX and 13-HPL pathway	0.00105	
	Divinyl ether biosynthesis II (13-LOX)	0.00105	
	Anandamide degradation	0.02291	
	Gibberellin inactivation	0.02291	
	IAA biosynthesis IV	0.02291	
	IAA biosynthesis VI (via indole-3-acetamide)	0.02291	
	Jasmonic acid biosynthesis	1.35E-06	
	Leucopelargonidin and leucocyanidin biosynthesis	0.00105	
	Nicotine degradation II	0.02291	
	Tetrapyrrole biosynthesis I	0.02291	
Nucleosides and nucleotide	ppGpp biosynthesis	0.02291	
	Salvage pathways of pyrimidine ribonucleotides	0.02291	
Amino acid and derivative metabolism	Asparagine biosynthesis I		0.00786
	Asparagine degradation I		0.00786
	Aspartate biosynthesis I		0.00786
	Aspartate degradation II		0.0095
	Cysteine biosynthesis I		0.00786
	formylTHF biosynthesis I		0.00095
	formylTHF biosynthesis II		0.00786
	Glutamine biosynthesis I		0.00786
	Glutamine degradation III		0.00786
	Glycine cleavage complex		0.00095
	Lysine biosynthesis I		0.00786
	Lysine biosynthesis II		0.00786
	Lysine biosynthesis VI		0.00786
	Threonine degradation II		0.00786
	Threonine degradation III (to methylglyoxal)		7.86E-03
Carbohydrates metabolism	Aminopropanol biosynthesis		0.00786
	Glycerol degradation I		0.00095
	Glycerol degradation IV		0.00786
	Glycolipid desaturation		0.00786
	Reductive TCA cycle I		7.86E-03
	Starch biosynthesis		0.00786
	Respiration (anaerobic)		0.00095
Cell Wall	Epicuticular wax biosynthesis		0.00786
	Homogalacturonan degradation		6.91E-08
	Suberin biosynthesis		0.00095
Lipid metabolism	Cyclopropane and cyclopropene fatty acid biosynthesis		0.00012
	Cyclopropane fatty acid (CFA) biosynthesis		0.00012
	Fatty acid biosynthesis—initial steps		7.40E-07
	Fatty acid elongation—saturated		6.91E-08
	Fatty acid elongation—unsaturated II		7.40E-07
	Phospholipid desaturation		0.00786
	Phospholipid biosynthesis I		0.00786
	Superpathway of fatty acid biosynthesis		6.91E-08
Nitrogen metabolism	Ammonia assimilation cycle II		0.00786
	Nitrate reduction II (assimilatory)		0.00786
Nucleosides and nucleotide	*De novo* biosynthesis of pyrimidine deoxyribonucleotides		0.00095
	Purine nucleotides de novo biosynthesis I		1.20E-04
	Purine nucleotides de novo biosynthesis II		0.00786
	Ribose degradation		0.00786
	Salvage pathways of purine nucleosides		0.00095
	Superpathway of ribose and deoxyribose phosphate degradation		0.00786
	tRNA charging pathway		5.06E-10
Photosynthesis	Chlorophyllide a biosynthesis		0.00095
Secondry metabolism	Chorismate biosynthesis		0.00095
	DIMBOA-glucoside degradation		0.00786
	Folate polyglutamylation I		0.00786
	Folate transformations		0.00095
	Phenylpropanoid biosynthesis		0.00786
	Phenylpropanoid biosynthesis, initial reactions		0.00786
	Salicylate biosynthesis		0.00786
	Secologanin and strictosidine biosynthesis		0.00012
Stress response	Removal of superoxide radicals		0.00786
Amino acid and derivatives metabolism	Arginine degradation I (arginase pathway)	0.02291	0.00095
	Glutamate degradation III	0.02291	0.00786
	Histidine biosynthesis I	0.02291	0.00095
	4-hydroxyproline degradation I	0.02291	0.00095
	Isoleucine biosynthesis from threonine	0.00105	0.00095
	Isoleucine degradation II	1.35E-06	0.00786
	Leucine degradation III	1.35E-06	0.00786
	Methionine biosynthesis II	0.02291	0.00095
	Methionine salvage pathway	0.02291	0.00786
	Phenylalanine degradation III	1.35E-06	0.00786
	Proline degradation I	0.02291	0.00095
	Proline degradation II	0.02291	0.00095
	Superpathway of leucine, valine, and isoleucine biosynthesis	0.00105	0.00095
	Superpathway of lysine, threonine and methionine biosynthesis II	0.02291	7.40E-07
	Threonine degradation III (to methylglyoxal)	0.02291	7.86E-03
	Tyrosine degradation I	0.02291	0.00786
	Valine biosynthesis	0.00105	0.00095
	Valine degradation I	0.00105	0.00786
	Valine degradation II	1.35E-06	0.00786
Carbohydrates metabolism	CALVIN cycle	2.97E-09	4.41E-09
	Cytokinins 7-N-glucoside biosynthesis	3.33E-05	6.41E-09
	Cytokinins 9-N-glucoside biosynthesis	3.33E-05	5.06E-10
	Cytokinins-O-glucoside biosynthesis	3.33E-05	5.06E-10
	Fructose degradation to pyruvate and lactate (anaerobic)	3.33E-05	4.88E-12
	Galactose degradation II	3.33E-05	6.91E-08
	Gluconeogenesis	1.35E-06	7.40E-07
	Glucose fermentation to lactate II	0.00105	6.91E-08
	Glycolysis I	1.35E-06	5.28E-11
	Glycolysis IV (plant cytosol)	3.33E-05	5.28E-11
	Mixed acid fermentation	0.000333	0.00095
	Pentose phosphate pathway (non-oxidative branch)	0.02291	0.00786
	Starch degradation	1.35E-06	0.00095
	Sucrose biosynthesis	0.002291	0.00786
	Sucrose degradation III	0.00105	0.00786
	Sucrose degradation to ethanol and lactate (anaerobic)	1.25E-10	3.43E-15
	UDP-galactose biosynthesis (salvage pathway from galactose using UDP-glucose)	0.00105	9.77E-06
	UDP-glucose conversion	0.02291	6.41E-09
	UDP-N-acetylgalactosamine biosynthesis	0.02291	9.77E-06
Cell wall	Cellulose biosynthesis	1.25E-10	4.02E-13
Co factors	Pantothenate and coenzymeA biosynthesis II	0.02291	0.00095
	Pantothenate biosynthesis I	0.02291	0.00095
	Pantothenate biosynthesis II	0.02291	0.00095
Energy metabolism and electron transmission	Aerobic respiration—electron donor II	1.35E-06	0.00095
	Aerobic respiration—electron donor III	3.33E-05	0.00095
	Aerobic respiration—electron donors reaction list	3.33E-05	7.40E-07
	NAD salvage pathway II	0.00105	7.40E-07
	NAD/NADH phosphorylation and dephosphorylation	3.33E-05	0.00012
	photorespiration	0.02291	0.00012
	Respiration (anaerobic)—electron donors reaction list	3.33E-05	7.40E-07
Lipid metabolism	Fatty acid β-oxidation I	1.35E-06	0.00786
	Fatty acid β-oxidation II (plant, saturated)	0.02291	0.00786
	Phospholipases	0.00105	0.00786
	Triacylglycerol degradation	3.33E-05	9.77E-06
Nucleosides and Nucleotides	Salvage pathways of purine and pyrimidine nucleotides	0.02291	0.00786
Plant hormone and Secondary metabolism	Ethylene biosynthesis from methionine	0.02291	0.00786
	Enterobactin biosynthesis	1.35E-06	0.00786
	Betanidin degradation	1.35E-06	1.06E-31
	Brassinosteroid biosynthesis II	3.33E-05	3.51E-14
Stress response	Glutathione-mediated detoxification	2.97E-09	0.00095

Pathway analysis of the 2371 DEGs showing similar responses to BPH in Bt and non-Bt rice plants using the Plant MetGenMAP system identified 145 significantly changed pathways with FDR correction at a threshold of 0.05: 43 pathways were significantly upregulated (raw values in yellow), while 52 pathways were significantly downregulated (raw values in light green). The remaining 59 pathways were either upregulated or downregulated.

### Profiles of DEGs in response to BPH infestation between Bt and non-Bt rice

A total of 439 probe-sets (representing 400 DEGs) showing differential responses to BPH infestation between the Bt and non-Bt rice were identified and classified into eight categories (Supplementary Table S3; Figure 1). There were 68.4% more upregulated genes in non-Bt parent than in Bt rice line, particularly genes involved in stress response, signal transduction, transcriptional regulation, transport, and unknown function (Figure 1B). Moreover, for most genes upregulated after BPH attack in both lines, FC values were more significant in non-Bt parent than in Bt rice. A high percentage of DEGs were in the categories genes of unknown function and stress response-related; 40.1% of DEGs (111, 121 probe-sets) were stress-related and were therefore further classified. As shown in Figure 1C, genes involved in oxidative stress response, pathogenesis-related proteins and protein inhibitors were significantly induced by BPH feeding, especially in non-Bt parent. In Bt rice, the expression of four of the 8 oxidative stress response genes remained unchanged, and two were even suppressed. Signal transduction-related genes and transcription factor (TF) genes were also more affected in non Bt parent than in Bt rice. The expression of 17 of the 20 TF genes was more significantly altered in non-Bt parent in response to BPH feeding, whereas that of three TF genes was only significantly altered in Bt rice, including the MYB (v-myb avian myeloblastosis viral oncogene homolog), TCP (Teosinte branched1/Cycloidea/ Proliferating cell factor 1) and bHLH (basic Helix-Loop-Helix) family TF genes (Figure 1; Supplementary Table S3). On the other hand, phytohormone biosynthesis and signaling genes were more affected by BPH feeding in Bt rice. GA biosynthesis and signaling-related genes were downregulated in Bt rice line but their expression remained unchanged in non-Bt parent; ET biosynthesis gene *ACS* (1-aminocyclopropane-1-carboxylate synthase) was more strongly suppressed while JA signaling-related genes were more strongly induced in Bt rice.

### Genes likely related to the altered performance of BPH fed on Bt rice

Genes specifically induced or repressed in Bt rice, and those that are more strongly induced or less repressed in Bt rice vs. the non-transgenic parent, are thought to be closely related to the altered performance of BPH. Of the 439 probe-sets showing differential expression in response to BPH between lines, 38 and 69 (representing 36 and 62 DEGs) were upregulated or downregulated, respectively, only in Bt rice (Figure 1A; Supplementary Table S4). Excluding two genes that showed similar expression patterns between rice lines in the absence of BPH treatment, 46 DEGs (47 probe-sets, 9 up/38 downregulated) with FC > 3.0 were considered most likely to contribute to the impact of Bt rice on BPH performance (Table 4). These DEGs included early nodulin genes, lipid metabolism genes, stress response genes and TF genes. *Early nodulin 93* (Os06g04990) and a retrotransposon gene (Os01g37350) were upregulated in non-transgenic parent but downregulated in Bt rice upon BPH feeding, whereas two lipid metabolism-related genes, *LTPL65* and *phosphotransferase*, were induced in Bt rice but repressed in the non-Bt rice plants. These four genes are also likely related to the variation in Bt rice (Table 4). Of the remaining 170 probe-sets showing significant changes in expression in both rice lines upon BPH attack, 14 (14 DEGs) were more significantly induced by BPH, while 50 (47 DEGs) were less suppressed in Bt rice. Excluding one showing similar expression patterns before BPH infestation, the remaining 60 DEGs (13 up/47 downregulated) probably participate in BPH-induced defense, including signal transduction-related genes, phytohormone biosynthesis and signaling genes and other stress response genes (Supplementary Table S4). Finally, 118 probe-sets were significantly upregulated and 40 were significantly downregulated only in non-transgenic parent; these might represent stress-sensitive genes.

**Table 4:** **Genes most likely contributing to the variation in BPH performance on Bt rice**.

**Probe set ID**	**BPH-infested/non-infested**				**Gene ID and annotation**	**Classification**
	**Bt**		**non-Bt**			
	**FC**	**RP**	**FC**	**RP**		
Os.50961.1.S1_at	4.125	D	1.046	–	LOC_Os03g58890//oxidoreductase, 2OG-Fe oxygenase family protein	Carbohydrates metabolism
Os.11244.3.S1_x_at	3.643	D	0.884	–	LOC_Os06g04200//Granule-bound starch synthase 1, chloroplast precursor	
Os.10546.1.S1_s_at	3.458	D	0.901	–	LOC_Os09g34230//UDP-glucoronosyl and UDP-glucosyltransferase family protein	
Os.21369.1.S1_at	3.383	D	1.740	–	LOC_Os08g32780//bifunctionalmonodehydroascorbate reductase and carbonic anhydrasenectarin-3 precursor	
Os.27281.1.S1_at	3.147	D	0.880	–	LOC_Os04g02620//oxidoreductase, short chain dehydrogenase/reductase family protein	
Os.49281.1.S1_at	3.025	D	0.926	–	LOC_Os06g21240//Glycine rich protein family protein	
OsAffx.27459.2.S1_s_at	9.600	D	0.685	–	LOC_Os06g05000//Early nodulin 93 ENOD93 protein	Growth regulation
Os.38638.3.S1_x_at	7.855	D	0.958	–	LOC_Os06g05010//Early nodulin 93, putative	
Os.38638.1.S1_at	5.593	D	2.311	U	LOC_Os06g04990//Early nodulin 93, putative	
Os.11212.1.S1_at	4.388	D	1.132	–	LOC_Os07g18750//LTPL42—Protease inhibitor/seed storage/LTP family protein precursor,	Lipids metabolm
Os.27520.1.S1_at	3.508	D	0.632	–	LOC_Os12g02320//LTPL12—Protease inhibitor/seed storage/LTP family protein precursor,	
Os.13246.1.S1_at	2.174	U	16.584	D	LOC_Os01g59870//LTPL65—Protease inhibitor/seed storage/LTP family protein precursor,	
Os.13835.2.S3_a_at	2.147	U	2.028	D	LOC_Os01g51920//phosphotransferase	
Os.9538.1.S1_s_at	3.415	U	1.344	–	LOC_Os06g39870//26S protease regulatory subunit 8	Nucleotides and protein metabolism
Os.27804.1.S1_at	4.783	D	0.629	–	LOC_Os08g10310//SHR5-receptor-like kinase	
Os.10246.4.S1_x_at	4.709	D	0.572	–	LOC_Os06g06510//Histone H3	
Os.16899.1.S1_at	3.617	D	0.718	–	LOC_Os07g30150//phosphoribosyl transferase	
Os.8570.3.S1_s_at	3.416	D	0.845	–	LOC_Os03g19600//retrotransposon protein, putative, Ty3-gypsy subclass	Others
Os.10255.1.S1_s_at	2.254	D	3.048	U	LOC_Os01g37350//retrotransposon protein, putative, Ty3-gypsy subclass	
Os.5044.1.S1_at	4.864	U	1.966	–	LOC_Os01g50410//STE_MEKK_ste11_MAP3K.6	Signal transduction
OsAffx.26237.1.S1_at	4.28	D	0.998	–	LOC_Os04g29770//wall–associated receptor kinase-like 3 precursor	
Os.12535.1.S1_at	6.262	U	1.769	–	LOC_Os01g52230//phosphoethanolamine/phosphocholine phosphatase	Stress response
Os.53670.1.S1_at	4.675	U	1.710	–	LOC_Os05g15880//glycosyl hydrolase	
Os.25329.1.A1_at	3.827	U	1.087	–	LOC_Os12g43440//Thaumatin-like protein precursor	
Os.20260.1.S1_at	6.624	D	1.475	–	LOC_Os01g22352//peroxidase 2 precursor	
Os.49627.1.S1_at	5.695	D	0.552	–	LOC_Os06g37150//L-ascorbate oxidase	
OsAffx.14201.1.S1_at	5.063	D	1.017	–	LOC_Os04g39360//heavy metal transport/detoxification protein	
OsAffx.32039.1.S1_x_at	5.013	D	0.518	–	LOC_Os12g35610//respiratory burst oxidase homolog	
Os.7611.1.S1_at	4.413	D	0.737	–	LOC_Os03g06670//Core histone H2A/H2B/H3/H4 domain containing protein	
Os.35510.1.S1_at	4.211	D	0.517	–	LOC_Os02g01220//Rhodanese-like domain containing protein	
Os.5338.1.S1_at	3.597	D	0.644	–	LOC_Os10g30150//universal stress protein family protein	
Os.5583.1.S1_at	3.328	D	1.242	–	LOC_Os03g19270//universal stress protein family protein	
OsAffx.11838.1.S1_x_at	3.154	D	1.0783	–	LOC_Os01g73250//abscisic stress-ripening	
Os.22580.1.S1_s_at	3.033	D	0.902	–	LOC_Os01g73250//abscisic stress-ripening	
Os.1479.1.S1_at	6.91	D	0.871	–	LOC_Os07g48980//Nicotianamine synthase 3	
Os.54454.1.S1_at	5.698	D	0.516	–	LOC_Os11g32650//chalcone synthase	
OsAffx.27442.1.S1_at	3.035	U	0.683	–	LOC_Os06g03670//dehydration-responsive element-binding protein 1A	Transcription factors
Os.21231.1.S1_at	6.147	D	0.600	–	LOC_Os01g38610//Helix-loop-helix DNA-binding domain containing protein	
Os.7512.1.S1_at	3.464	U	1.698	–	LOC_Os04g56990//myb-like DNA-binding domain, SHAQKYF class family protein	
Os.9303.1.S1_at	4.955	D	0.559	–	LOC_Os02g46460//peptide transporter PTR3-A	Transport
Os.57361.1.S1_at	7.482	U	1.948	–	LOC_Os08g13400//hypothetical protein	Unknown
Os.9886.1.S1_at	3.544	U	1.227	–	LOC_Os04g02530//Conserved hypothetical protein	
Os.56964.1.S1_at	6.937	D	0.912	–	LOC_Os06g46980//expressed protein	
Os.5390.1.S1_at	6.669	D	0.582	–	LOC_Os12g33130//expressed protein	
Os.8558.1.S1_at	4.388	D	0.629	–	LOC_Os02g11770//hypothetical protein	
OsAffx.23641.1.S1_at	3.635	D	0.595	–	LOC_Os01g43230//expressed protein	
OsAffx.31409.1.S1_s_at	3.558	D	1.314	–	LOC_Os11g40660//hypothetical protein	
Os.50018.1.S1_at	3.475	D	0.623	–	LOC_Os07g47750//expressed protein	
Os.53428.1.S1_at	3.391	D	0.600	–	LOC_Os09g26370//expressed protein	Unknown
Os.7382.1.S1_at	3.274	D	0.939	–	LOC_Os05g46950//expressed protein	
OsAffx.16877.1.S1_at	3.117	D	0.795	–	LOC_Os08g07490//expressed protein	

Nine Bt rice-specific upregulated and 37 (38 probe-sets) Bt rice-specific downregulated genes, as well as four genes showing opposite responses to BPH between Bt and non Bt rice plants were identified. These genes are involved in carbohydrate, lipid, nucleotide and protein metabolism, growth regulation, signal transduction, stress responses, or they encode transcription factors and transporters. FC, fold change value; RP, regulation pattern; D, down regulated; U, up regulated; –, no significant change.

### Quantitative RT-PCR verification of genes contributing to the effect of Bt rice on BPH performance

**Figure 2:**
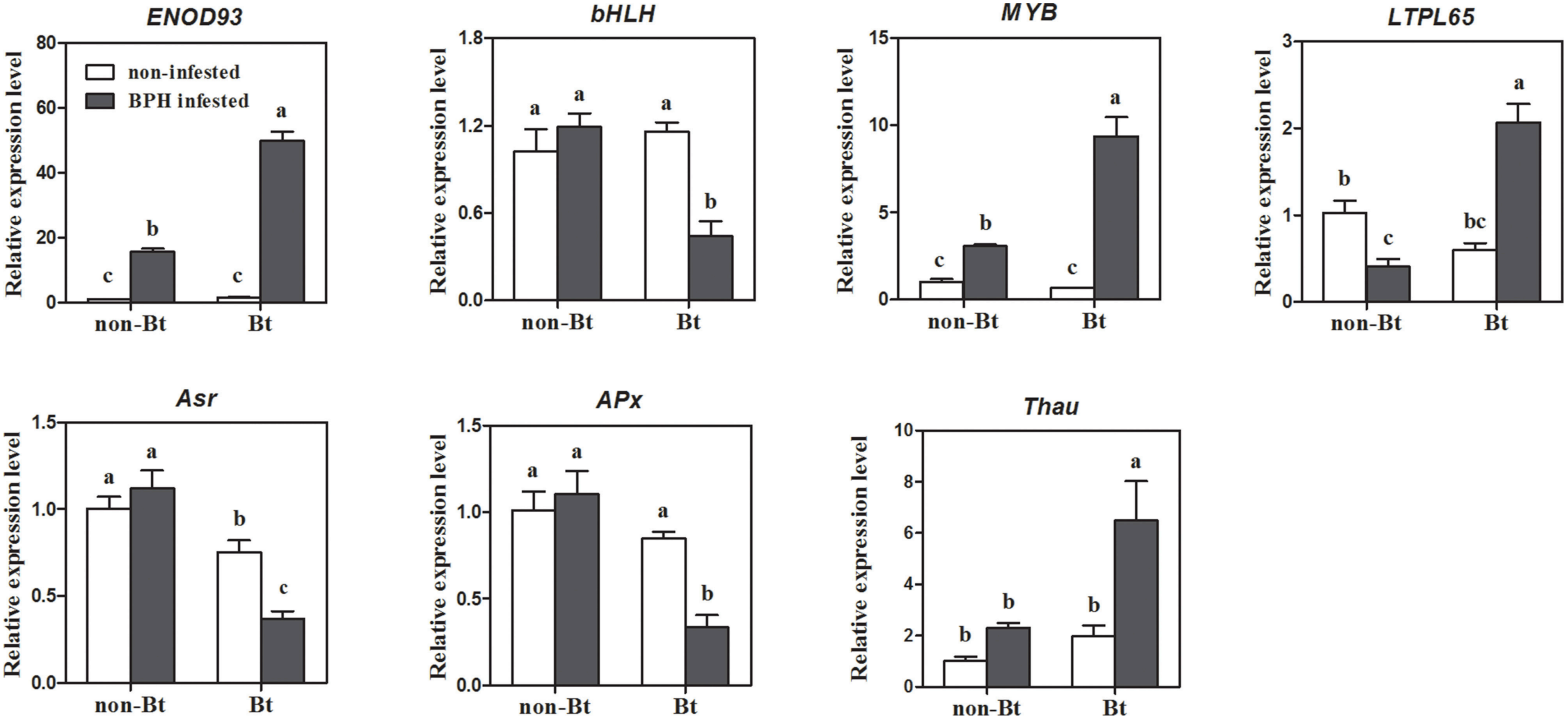
**Quantitative RT-PCR verification of seven genes likely involved in the variation of BPH performance on Bt rice**. *ENOD93, early nodulin 93* (LOC_Os06g04990); *bHLH, Helix-loop-helix DNA-binding domain containing protein* (LOC_Os01g38610); *MYB, myb-like DNA-binding domain, SHAQKYF class family protein* (LOC_Os04g56990); *LTPL65, protease inhibitor/seed storage/LTP family protein* (LOC_Os01g59870); *Asr, aba stress-ripening* (LOC_Os01g73250); *APx, L-ascorbate oxidase* (LOC_Os06g37150); *Thau, thaumatin-like protein* (LOC_Os12g43440). Error bars represent SD values (*n* = 3); different letters indicate significant differences (*P* < 0.05).

We selected seven genes for qRT-PCR verification out of the 50 DEGs (51 probe-sets) that most likely contribute to the effect of Bt rice on BPH performance, including two TF genes, lipid metabolism gene *LTPL65*, early nodulin gene *ENOD93* (Os06g04990), ABA-responsive gene *Asr* and two other stress response genes. As shown in Figure 2, the expression patterns of these seven genes were almost entirely consistent with the data obtained from microarray analysis, except for *ENOD93*. TF gene *bHLH* (Os01g38610) was specifically repressed, while *MYB* (Os04g56990) was specifically induced in Bt rice line. ABA responsive gene *Asr* (Os01g73250) and L-ascorbate oxidase *APx* (Os06g37150) were specifically downregulated upon BPH attack, while a pathogenesis-related thaumatin-like protein gene (Os12g43440) was specifically induced in Bt rice. The qRT-PCR analysis also confirmed the opposite expression patterns of *LTPL65* between Bt rice and non-Bt parent. However, the expression of *ENOD93* was dramatically increased in Bt rice upon BPH feeding, as revealed by qRT-PCR analysis, which contrasts with the results of microarray analysis.

### Verification of the involvement of phytohormones in induced BPH defense

**Figure 3:**
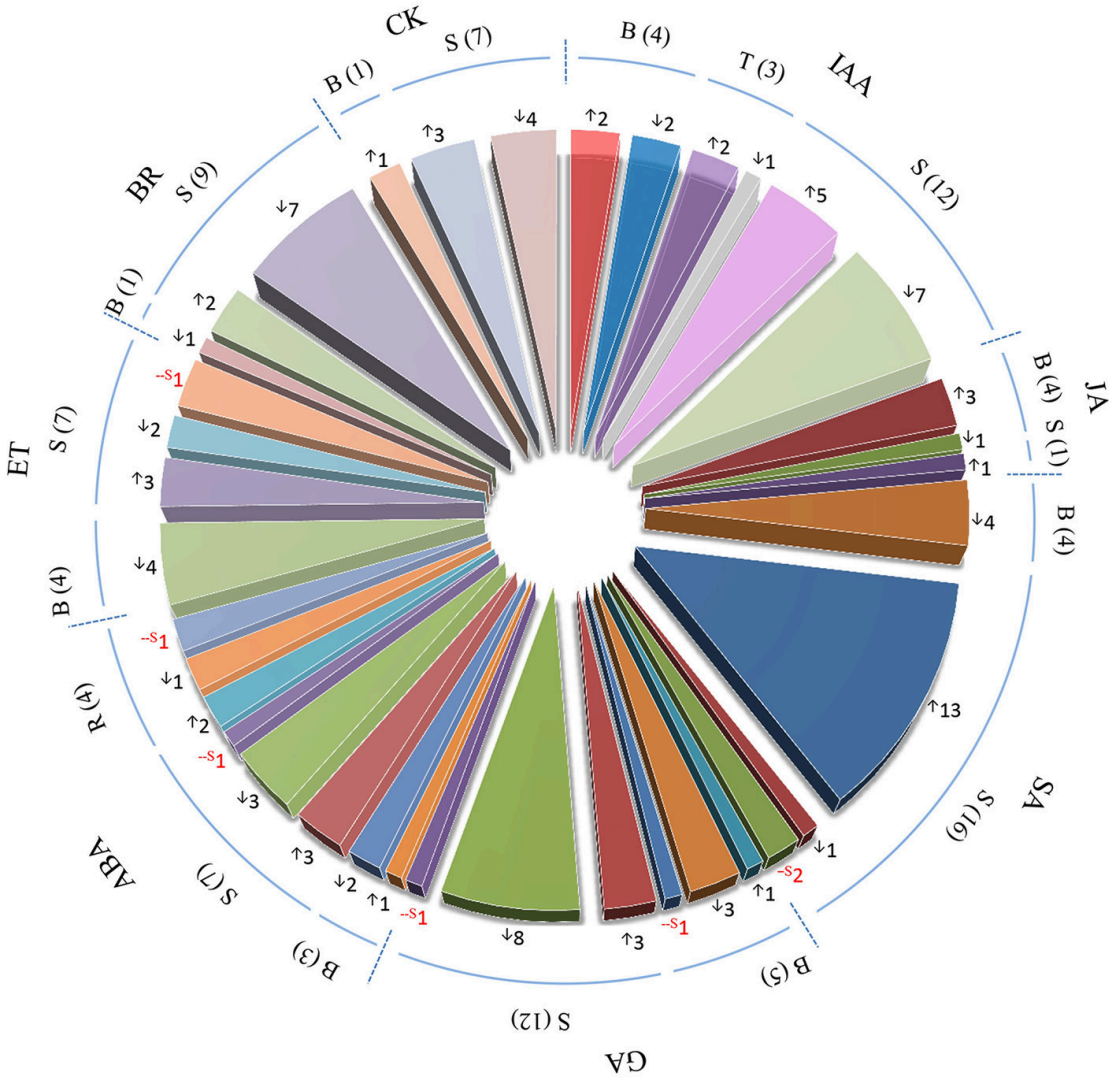
**Number of phytohormone biosynthesis, transport, and signaling-related genes identified by microarray analysis in response to BPH infestation**. IAA, indole-3-acetic acid; JA, jasmonic acid; SA, salicylic acid; GA, gibberellins; ABA, abscisic acid; ET, ethylene; BR, brassinosteroid; CK, cytokinin. B, biosynthesis-related genes; S, signaling-related genes; R, responsive genes; T, transport-related genes. Numbers in brackets and beside arrows indicate the numbers of phytohormone biosynthesis-, transport- or signaling-related genes. ^↑^upregulated in both rice lines; ^↓^downregulated in both rice lines; -S in red color, significant change was only detected in one rice line.

Pathway analysis of genes with similar expression patterns revealed that IAA and JA biosynthesis, and GA deactivation pathways were induced upon BPH feeding, while SA biosynthesis was suppressed. Analysis of DEGs also suggested that hormone biosynthesis and signaling genes probably participate in BPH-induced defense (Figure 3; Supplementary Table S5). Therefore, we analyzed the expression patterns of hormone biosynthesis and signaling genes, along with the endogenous concentrations of phytohormones including IAA, JA, GA, SA, and ABA.

**Figure 4:**
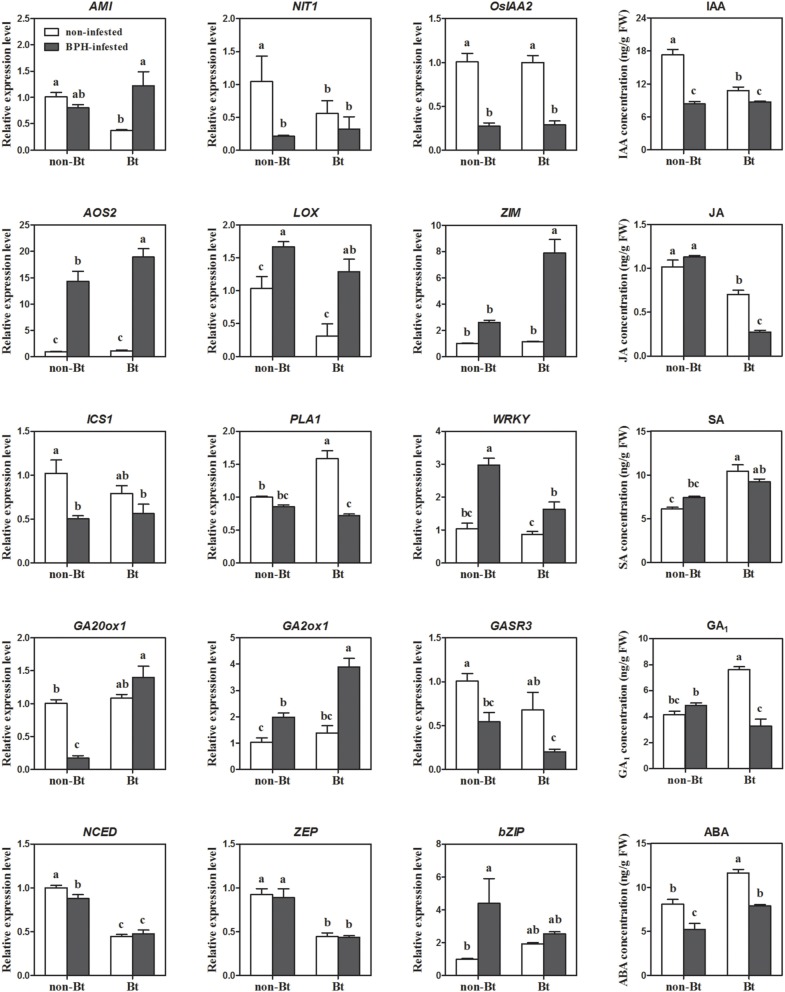
**Expression levels of phytohormone biosynthesis and signaling genes revealed by qRT-PCR, and endogenous IAA, JA, GA, SA, and ABA levels in Bt and non-Bt rice in response to BPH infestation**. *AMI, amidase* (LOC_Os04g10530); *NIT, nitrilase-associated protein* (LOC_Os04g48870); *OsIAA2, Auxin-responsive Aux/IAA gene family member* (LOC_Os01g09450); *AOS2, allene oxide synthase 2* (LOC_Os03g12500); *LOX, lipoxygenase* (LOC_Os08g39850); *ZIM, ZIM motif family protein* (LOC_Os03g08320); *ICS1, isochorismate synthase 1* (LOC_Os09g19734); *PAL, phenylalanine ammonia-lyase* (LOC_Os04g43760); *WRKY, WRKY 2* (LOC_Os03g33012); *GA20ox1, gibberellin 20 oxidase 1* (LOC_Os03g63970); *GA2ox1, x/IAA gibberellin 2-oxidase 1* (LOC_Os05g06670); *GASR3, Gibberellin-regulated GASA/GAST/Snakin family protein* (LOC_Os03g55290); *NCED, 9-cis-epoxycarotenoid dioxygenase* (LOC_Os02g47510); *ZEP, zeaxanthin epoxidase* (LOC_Os04g37619); *bZIP, bZIP transcription factor family protein* (LOC_Os02g09830). Error bars represent SD values (*n* = 3); different letters indicate significant differences (*P* < 0.05).

Four IAA biosynthesis-related DEGs were identified by microarray analysis. Upon BPH infestation, *indole-3-glycerol phosphate synthase (IGS)* and *amidase (AMI)* were upregulated, while *nitrilase (NIT)* and *IAA-amino acid hydrolase* were downregulated. All auxin-responsive *IAA/AUX* and *ARF* genes were downregulated except *OsIAA18*, which might have resulted from the reduced levels of IAA after BPH feeding (Figure 4; Supplementary Table S5). Meanwhile, most *SAUR* genes were induced. IAA concentrations were more significantly reduced in non-Bt parent (in which *NIT1* was markedly suppressed upon BPH feeding) than in Bt rice line. *AMI* was significantly induced in Bt rice line, while a slight decrease in expression was revealed in non Bt parent by qRT-RCR analysis, which is also in accordance with the change in IAA levels (Supplementary Table S5; Figure 4). Three of four JA biosynthesis pathway genes and one JA signaling gene were upregulated in both rice lines in response to BPH infestation, which is consistent with the qRT-PCR results. However, the endogenous JA levels did not significantly increase, and it even decreased in Bt rice (Figures 3, 4). Microarray analysis revealed that the expression of bioactive GA biosynthesis genes *GA20ox1* and *GA20ox2* was suppressed by BPH attacking, and a more severe effect was found in Bt rice. GA inactivation gene *GA2ox1* was upregulated, while *GA2ox3* was downregulated, in response to BPH infestation, whereas most of the predicted GA receptor genes (such as *GID1L2*) were downregulated. In non-transgenic parental plants, GA levels showed no obvious changes, even though the reduced *GA20ox1* expression and inducted *GA2ox1* expression were verified by qRT-PCR. Significant reductions in GA_1_ levels were found in Bt rice line, although no significant change in *GA20ox1* was detected by qRT-PCR (Supplementary Table S5; Figure 4). BPH attack did not alter SA concentrations in either line, although the expression of *phenylalanine ammonia-lyase (PAL)* and *isochorismate synthase 1 (ICS1)* was suppressed. On the contrary, ABA concentrations were significantly reduced upon BPH infestation, but ABA biosynthesis-related genes did not show significant changes in expression, except for Mo-cofactor. In addition, 14 SA signaling-related WRKY TF genes were significantly induced, while ABA signaling and -responsive genes were either up- or downregulated upon BPH infestation (Supplementary Table S5; Figures 3, 4). As indicated by microarray analysis, ET and BR biosynthesis-related genes were suppressed in both rice lines, while a cytokinin (CK) deactivating enzyme gene was upregulated upon BPH infestation. ET and CK signaling-related genes were either up- or downregulated in both rice lines. Seven of 9 BR signaling-related *BAK1* genes *(Brassinosteroid insensitive 1-associated receptor kinase 1)* were suppressed in both Bt and non-Bt rice plants, while one (Os11g31540) was dramatically induced in the non-Bt parent. In addition, significant changes in the expression of genes encoding AP2 domain-containing proteins were only detected in non-Bt parent.

## Discussion

In this study, we determined that the general response to BPH infestation is similar in Bt rice KMD2 vs. non-Bt parent Xiushui 11, as only approximately 10% of genes exhibited differential expression patterns. According to the results of pathway analysis, inhibition of chlorophyll biosynthesis, nitrogen assimilation, lipid metabolism and amino acid biosynthesis was detected in both rice lines. Meanwhile, IAA and JA biosynthesis and GA deactivation pathways were induced (Table 3). The expression of genes encoding protein inhibitors, pathogen-related proteins and other stress response genes was induced in response to BPH infestation, which is similar to many biotic and abiotic stress responses (Figure 1). Indeed, continuous ingestion of phloem sap by BPH reduces plant growth by inducing leaf senescence and disrupting photosynthesis. Suppression of genes involved in photosynthesis and cell growth by BPH was also detected in Minghui63 (Yuan et al., 2005). A shift from basic metabolism to defense responses appears to be a common strategy used by plants suffering from biotic and abiotic stress.

The defense response of plants to piercing-sucking pests resembles the response to pathogens (Goggin, 2007; Wang et al., 2012a). As explained above, PTI, together with the effectors secreted in watery saliva, promote the basal resistance response, including the activation of Ca^2+^ influx as well as phytohormone and MAPK cascade signaling pathways. In the current study, Ca^2+^ signaling appeared to function in the BPH response, as a series of *calmodulin* genes were upregulated in response to infestation. Ascorbate peroxidase (APx) and most peroxidase (POD) family genes (21) were suppressed in both rice lines, which might result in the accumulation of H_2_O_2_ as a second messenger (Supplementary Table S2; Figure S1). By contrast, seven *POD* genes were induced after BPH attacking, especially in non-Bt parent, which suffered heavier oxidative stress (Supplementary Table S3; Figure S1). The induction of *POD* is thought to be required for the scavenging of excessive ROS.

Many studies have demonstrated that JA, SA, and ET are involved in BPH resistance. However, this effect is positive or negative remains controversial. The present results show that in response to BPH exposure, JA biosynthesis was activated while SA and ET biosynthesis was suppressed. A similar result was reported by Wei et al. (2009), who suggested that JA biosynthesis-related genes are induced by the wounding caused by BPH. However, in the current study, JA and SA levels were not significantly altered upon BPH attack, except for a reduction in JA levels in the Bt rice line. Meanwhile, all JA and SA signaling-related genes were activated after BPH infestation (Figure 4; Supplementary Table S5). Auxin, ABA and GAs have been shown to be involved in defense responses to aphid feeding (Divol et al., 2005; Park et al., 2006). ABA and IAA signaling were proposed to be associated with the rice–BPH interaction (Zhang et al., 2004). The present results show that IAA and GA levels decreased as a result of the repression of biosynthesis-related genes or the induction of inactivation-related genes. In accordance with these observations, a number of signaling genes were downregulated in response to BPH. Similar results were obtained for BR, another regulator of plant growth and development. Therefore, the reduced growth of rice plants under BPH attack might be regulated by IAA, GA, and BR. A recent study revealed that WRKY70 is involved in the trade-off between defense and growth through regulating JA and GA biosynthesis (Li et al., 2015b). In the current study, ABA concentrations decreased significantly in both rice lines in response to BPH attack, although ABA biosynthesis genes showed no difference in expression. Mo-factors, as well as ABA signaling and responsive genes, were either down- or upregulated in response to BPH attack. Meanwhile, *Asr* (Os01g73250) was specifically suppressed in Bt rice plants (Supplementary Table S5; Figure 2). Extensive feeding by phloem feeders is thought to trigger water stress and senescence, which alters the expression of ABA-induced genes (Divol et al., 2005). Based on our results, we conclude that phytohormones play a role in balancing plant growth and defense responses in plants under stress conditions. The growth related hormonrs are sensitive to herbivore attack. Expression of growth-related hormone signaling genes changed via feedback regulation. Thus, the shift from growth to defense is started in BPH-infested plants. Subsequently, defense-related hormone signaling pathways, such as the JA, SA, and ABA signaling pathways, directly regulate defense/resistance genes and, consequently, the levels of defense-related compounds. In addition, IAA- GA-, and BR-mediated signaling might also participate in induced BPH defense, as crosstalk among plant hormones commonly occurs in most biological processes. JA, ABA, and ET interact with GA signaling by modulating the levels of DELLA repressors or *ent*-kaurene synthase A (Achard et al., 2007; Zentella et al., 2007; Qi et al., 2011; Yang et al., 2012, 2013). The GAST family gene *OsGSR1* activates BR biosynthesis by directly regulating a BR biosynthetic enzyme (Wang et al., 2009).

We detected 400 genes with differential responses to BPH between Bt rice and non-Bt parent plants, which suggests that some variation associated with the plant–BPH interaction might have occurred during plant transformation. We investigated 50 DEGs that probably contribute to the changes that render Bt rice less suitable for BPH consumption, including three early nodulin genes, four lipid metabolic genes, 14 stress response genes, three TF genes and genes with other functions. Nodulins were first recognized as a group of proteins induced by *Rhizobium* infection in the root nodules of leguminous plants (Legocki and Verma, 1980; Govers et al., 1985). *OsENOD93*, which was first isolated from rice by Reddy et al. (1998), is highly expressed in roots and suspension-cultured cells without elicitor. The identification of nodulin-like genes in non-nodulating plants suggests a possible role for nodulin-like proteins in regulating plant growth and development, although the functions of most nodulin-like proteins remain unclear. Recent studies have highlighted the transporter activity of nodulin-like proteins (Denancé et al., 2014). Members of the early nodulin-like (ENODL) family are related to phytocyanin, but they lack amino acid residues for copper binding (Mashiguchi et al., 2009). A phytocyanin-related early nodulin-like gene from *Boea crassifolia, BcBCP1*, increases osmotic tolerance in transgenic tobacco (Wu et al., 2011). The *ENOD93* gene identified in the present study encodes a protein with two transmembrane domains and the conserved ENOD domain, which might be involved in carbohydrate transport, as proposed by Chen (2014). This gene was induced by BPH infestation more strongly in Bt rice than in non-Bt parent plants, as revealed by qRT-PCR, which contrasts with the results of microarray analysis. Whether this gene is involved in BPH defense requires further study.

Plant non-specific lipid transfer proteins (nsLTPs) transport phospholipids, as well as glycolipids, across membranes. The antimicrobial activity of nsLTPs was first discovered by screening plant extracts that inhibit the growth of pathogens *in vitro*. LTPs isolated from the leaves of barley, maize, Arabidopsis and spinach (*Spinacia oleracea*) have antimicrobial activity against the bacteria *Clavibacter michiganensis* subsp. *sepedonicus* and *Ralstonia solanacearum* and the fungus *Fusarium solani* (Molina et al., 1993; Segura et al., 1993). Rice LTP expressed in *Escherichia coli* has activity against *Pyricularia oryzae* and the bacterium *Pseudomonas syringae*, and it delays the growth of *Xanthomonas oryzae* (Ge et al., 2003). In addition to the pathogen response, *nsLTP* genes are also regulated by abiotic stress in maize (*Zea mays*) and wheat (*Triticum aestivum* L.) (Jang et al., 2004; Wei and Zhong, 2014). Therefore, plant nsLTPs are thought to play an important role in plant defense. In the present study, 10 *LTP* genes were regulated by BPH infestation, nine of which showed differential responses to BPH damage between rice lines. *LTPL159* and *LTPL82* (encoding 2S albumin storage protein according to Boutrot et al., 2008) were more highly induced in non-Bt parent. The expression of four *LTPL* genes was more significantly reduced in Bt rice than in non-Bt parent. *LTPL65* was repressed in the non-Bt parent but significantly upregulated in Bt rice plants. Therefore, we speculate that LTPs are involved in the BPH defense response, especially LTPL65. The hypothesis that LTPs are involved in plant systemic resistance signaling was previously proposed by Maldonado et al. (2002). Buhot et al. (2004) revealed that tobacco (*N. tabacum*) LTP1 can bind to JA, and formation of the LTP–JA complex facilitates its recognition by elicitin receptors, thus inducing long distance protection against *Peronospora parasitica*. Arabidopsis AZI1, an LTP-related hybrid proline-rich protein, was identified as a novel target of MPK3, which is involved in salt stress signaling (Pitzschke et al., 2014). Moreover, major allergens in *Asparagus officinalis, B. oleracea* var. *capitata* and *Zea mays* are LTP family proteins (van Ree, 2002; Palacín et al., 2006; Carvalho and Gomes, 2007). Rice LTPL65 has a glycosyl phosphatidylinositol (GPI)-anchor in addition to its eight cysteine motif backbone, which helps this protein attach to the exterior side of the plasma membrane. Therefore, this protein is more like a signaling component than an allergen. The exact role of nsLTPs in BPH defense remains to be determined.

TFs are protein complexes that can help RNA polymerase bind to specific DNA sequences, thereby controlling the rate of gene transcription. WRKY genes have been implicated in multiple biotic and abiotic stress responses (Barah et al., 2013; Wang et al., 2013). In the present study, all WRKY genes responsive to BPH attack were upregulated, especially in non-Bt parent plants. WRKY genes are also induced by cabbage aphid attack in Arabidopsis, whereas they are repressed by both aphid and whitefly attack in cotton (Kusnierczyk et al., 2008; Dubey et al., 2013). Silencing of *SlWRKY70* attenuates *Mi-1*-mediated resistance against potato aphid and root-knot nematode, showing that *SlWRKY70* is required for *Mi-1* function (Atamian et al., 2012). NAC, MYB, and zinc finger TF family members are primarily responsive to pathogen infection and abiotic stress (Huang et al., 2007; Xia et al., 2010; Liu et al., 2011; Deng et al., 2012; Sun et al., 2012). AP2, NAC, and zinc finger family TFs, together with WRKY, are thought to be stress sensitive or involved in inducible defense responses, as more significant responses were detected in the more severely affected non-Bt parent. Meanwhile, *MYB* (Os04g56990) was induced upon BPH infestation, especially in Bt rice. One bHLH family TF gene was specifically repressed in Bt rice line (Os01g38610), while another was specifically repressed in non-transgenic parent plants (Os04g49450). The two TF genes that showed more significant responses in Bt rice (*bHLH* [Os01g38610] and *MYB* [Os04g56990]) are considered to represent candidate genes involved in the variation in Bt rice related to its impact on BPH performance. MYB and bHLH family TF genes were also identified as constitutive BPH resistance genes by Wang et al. (2012b). MYB TF is thought to function in reallocating energy to enhance defense responses, as several members of this gene family play important roles in photosynthesis and related metabolism (Saibo et al., 2009). R2R3-MYB and bHLH type TFs are also involved in the phyenylpropanoid pathway through regulating the biosynthesis of anthocyanin (Schwinn et al., 2014). It was recently demonstrated that plants prioritize defense over growth through regulation by WRKY. Moreover, an R2R3-type MYB TF, NaMYB8, modulates the accumulation of phenylpropanoid polyamine conjugates, which are involved in herbivore defense (Kaur et al., 2010). Identifying the targets of the candidate TFs requires further study.

## Conclusion

We compared the expression profiles of Bt rice vs. its non-transgenic parent in response to BPH infestation, as a previous study revealed significantly longer nymphal developmental duration and lower fecundity in BPH fed on KMD2. Basic metabolism, as well as growth-related hormone biosynthesis and signaling, were inhibited in response to BPH attack, while defense-related hormone signaling was induced. Based on our results, we conclude that phytohormone signaling play an important role in the shift form plant growth to defense in plants under stress conditions. Further studies on the crosstalk between growth-related hormone signaling and defense-related hormone signaling may come to be a key to understand the mechanism of plants' fight against biological or abiological stresses.

We found that 10% of genes showed differential responses to BPH between Bt rice and its non Bt parent, including 50 DEGs that are likely related to the impact of Bt rice on BPH performance. Among these, the early nodulin gene *ENOD93* and non-specific lipid transfer protein gene *LTPL65*, as well as two TF genes, are considered to represent candidate genes that contribute to the enhanced defense of Bt rice to BPH. Whether these genes could be used to improve rice BPH resistance remains to be investigated.

## Author contributions

FW, YC, and GY conceived and designed and performed the experiments. FW and YC performed the microarray analysis and analyzed the data. FW, DN, CD, NH, and YL contributed to the preparation of experiment materials, and qRT-PCR, LC-MS analysis. FW wrote the manuscript.

## Funding

This work was supported by the National Special Transgenic Project from the Chinese Ministry of Agriculture (2014ZX08011-001), and China National Science Fund for Innovative Research Group of Biological Control (Grant No. 31321063).

### Conflict of interest statement

The authors declare that the research was conducted in the absence of any commercial or financial relationships that could be construed as a potential conflict of interest.
