# Supplementary material for: Comparing Gene Expression Profiles Between Bt and non-Bt Rice in Response to Brown Planthopper Infestation
Source: Front Plant Sci. 2015 Dec 24;6:1181. doi: 10.3389/fpls.2015.01181 (PMC4689863; doi:10.3389/fpls.2015.01181)
Supplement: Supplementary file 1 [file Table1.DOCX]

| **Gene** | **ID** | **Primer** |
| --- | --- | --- |
| *ENOD93* | LOC_Os06g04990 | F: 5’-AACATTGCAGGGGTTAAGGC, R: 5’-GCCAGCGAGAGGATCTTCTT |
| *LTPL65* | LOC_Os01g59870 | F: 5’-AACATGTCGGACTGCCTGA, R: 5’-GCTTGTAGTCGACGCTGATG |
| *bHLH* | LOC_Os01g38610 | F: 5’-CAACTACCTCCGCTTCCTCA, R: 5’-GATCAGCTGCTCGACTCCTC |
| *MYB* | LOC_Os04g56990 | F: 5’-GTACATGCCAGCGTCATCTG, R: 5’-GCTTGTAGTCGACGCTGATG |
| *L-APx* | LOC_Os06g37150 | F: 5’- CAGCGTCACCATGAACAACA, R: 5’- AGTGGCCGTGGTAGAAGTAG |
| *Thaumatin* | LOC_Os12g43440 | F: 5’-GAGTTCACCGACCGTCTCAAGG R: 5’-TTCATCTCGTCAAGAAGCCCAG |
| *Asr6* | LOC_Os01g73250 | F: 5’- ACTACTCCAGCACCGTGGAC, R: 5’- GCTGCCTCATCAGGTACTCC |
| *Actin* | LOC_Os03g50885 | F:5’-TGTATGCCAGTGGTCGTACCA, R: 5’-CCAGCAAGGTCGAGACGAA |
| *Amidase* | LOC_Os04g10530 | F:5’-CAAAGGCAAGAGGATTGGTATT, R: 5’-TATGACATCTGCAAGGGAACG |
| *Nitrilase* | LOC_Os04g48870 | F:5’-CACCCCGGCCACTCTAGATA, R: 5’-GATAGCCGCCAACAAAAGCT |
| *OsIAA2* | LOC_Os01g09450 | F:5’-CTTCTCCTCCTCCTCCTCCT, R: 5’-TCTTTGCTCGCCTTGTCGC |
| *AOS2* | LOC_Os03g12500 | F:5’-CAATACGTGTACTGGTCGAATGG, R:5’-AAGGTGTCGTACCGGAGGAA |
| *LOX5* | LOC_Os08g39850 | F:5’-GCAGGGTAGGCAGCGAGTTGT, R: 5’-TTCTTCCCTGTCTTCGCTTCAA |
| *ZIM* | LOC_Os03g08320 | F:5’-TGAAGGAGCACAGTGGAAGC, R: 5’-CTTGCGTGTCTTTCAGCGTC |
| *ICS1* | LOC_Os09g19734 | F:5’-TATGGTGCTATCCGCTTCGAT, R: 5’-CGAGAACCGAGCTCTCTTCAA |
| *PAL1* | LOC_Os04g43760 | F:5’-GGGCAACCCAGTGACCAA, R: 5’-CGATTGCCTCGTCGGTCTT |
| *WRKY* | LOC_Os03g33012 | F:5’-AGAACAGGGCTCCCAAGATT, R: 5’-TTTTGTTCGCATTTCCATCA |
| *GA20ox1* | LOC_Os03g63970 | F:5’-GCTGTCGTTCCGGTACTCAT, R: 5’-AAGAATCGCCGGAAGTAGTG |
| *GA2ox1* | LOC_Os05g06670 | F:5’- GCTTACCACAAACGCTGACA , R: 5’-TGTAGCCCTTCCACATCGTT |
| *GASR3* | LOC_Os03g55290 | F:5’-AAGCTCAACACCACCACCAC, R: 5’-TCTGTTCGTCGTCCGTGAAG |
| *NCED* | LOC_Os02g47510 | F:5’-CTCACCATGAAGTCCATGAGGCTT, R: 5’-GTTCTCGTAGTCTTGGTC |
| *ZEP* | LOC_Os04g37619 | F:5’-GGATGCCATTGAGTTTGGTT, R: 5’-TGGCTGACTGAAGTCTCTCG |
| *bZIP* | LOC_Os02g09830 | F:5’-CCCAGCTCAGAGAAGAAGCT, R: 5’-TAATGTTCGTGGTGCTGCAG |

Supplementary Table S1. Primers used for qRT-PCR analysis.
