# Supplementary figures and images for: Comparing Gene Expression Profiles Between Bt and non-Bt Rice in Response to Brown Planthopper Infestation
Source: Front Plant Sci. 2015 Dec 24;6:1181. doi: 10.3389/fpls.2015.01181 (PMC4689863; doi:10.3389/fpls.2015.01181)

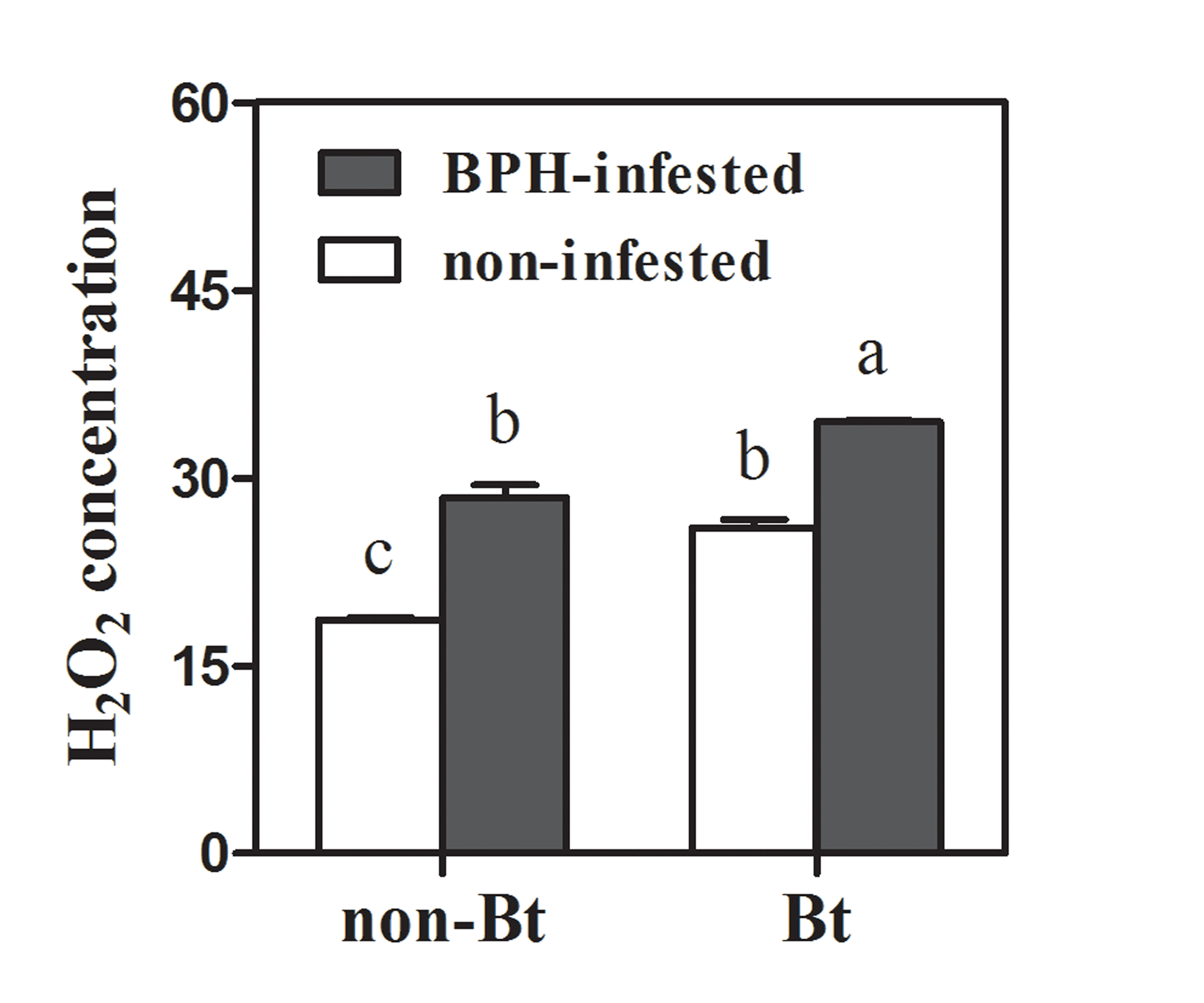

Supplement: Supplementary file 6 [file Image1.TIF]
